# The complete mitochondrial genome of *Citrus sinensis*

**DOI:** 10.1080/23802359.2018.1473738

**Published:** 2018-05-15

**Authors:** Fengwen Yu, Changwei Bi, Xuelin Wang, Xin Qian, Ning Ye

**Affiliations:** a College of Information Science and Technology, Nanjing Forestry University, Nanjing, Jiangsu, China;; b School of Biological Science and Medical Engineering, Southeast University, Nanjing, Jiangsu, China;; c Housing and Real Estate Promotion Center of Jiangsu Provincial Department of Housing and Urban-rural Development, Nanjing, Jiangsu, China

**Keywords:** *Citrus sinensis*, mitochondrial genome, phylogeny

## Abstract

*Citrus sinensis* is an important agricultural product with huge economic value. In this study, we report the complete mitochondrial (mt) genome sequence of *Citrus sinensis* for the first time. Our assembly of the *Citrus sinensis* mt genome resulted in a final sequence of 640,906 bp in length which contains 63 genes. Phylogenetic analysis showed that *Citrus sinensis* was closely related to *Brassica napus* and *Arabidopsis thaliana*, which also belong to malvids. The complete mt genome will facilitate further genetic studies of *Citrus sinensis*.

*Citrus sinensis*, also known as sweet orange, is an important agricultural product of immense economic value. *Citrus sinensis* is a member of Rutaceae. The genus *Citrus* includes five major cultivated species, including *Citrus sinensis* (sweet orange), *Citrus reticulata* (tangerine and mandarin), *Citrus limon* (lemon), *Citrus grandis* (pummelo), and *Citrus paradisi* (grapefruit) (Xu et al. 2013). Moreover, oranges are significant nutritional source for human health.

The whole genomic DNA was extracted from leaves of a mature sweet orange tree which was growing in a citrus orchard in Dundee, Florida (28°01’N 81°37’W). Genome sequence was generated on 454 platforms. Fourteen different single-end shotgun libraries were prepared as well as several paired-end libraries with pair distances of 3 kb and 8 kb for sequencing on the 454 platform to ensure that coverage of different parts of the genome was as even as possible. A total of 51.5× of 454 sequencing data were generated (Wu et al. 2014). Mitochondrias are essential organelles in plants which produce the energy of the cell. In this study, we described the assembly and annotation details of the *Citrus sinensis* mt genome, which will provide help to the study of molecular identification, genetic diversity, and phylogenetic classification in Rutales. The complete mitochondrial (mt) genome was submitted to the GenBank under the accession number of NC_037463.

All the 454 GS FLX titanium raw reads were assembled using Newbler 2.7. The original contigs generated by Newbler were a mixture of nuclear and organellar DNA. For the purpose of isolating mitochondrion contigs from all the contigs we obtained, we used a method described by Wang et al. (2018). According to Wang’s method, the coverage between 10 and 100 were preliminary set to filter the most likely mt contigs out. To visualize the connections among the filtered out mt contigs, we used Perl scripts and a text file named ‘454ContigGraph.txt’ which was generated by Newbler. Referring to the reads depth of the contigs and the connecting map, we removed false links and wrong forks manually. After this, a circular mt genome was developed.

The complete *Citrus sinensis* mt genome is 640,906 bp in length. The overall G + C content of mt genome is 43.89%. The physical map of *Citrus sinensis* mt genome was generated with OGDRAW (Lohse et al. 2007). With the help of the online program MITOFY, a total of 63 genes were identified, including 32 protein-coding genes, 28 tRNAs, and three rRNAs. Most of these genes were single copy genes, while five genes existed as double copies, including one protein-coding gene (rpl2) and four tRNA genes (trnl-CAU, trnA-Val, trnA-Asp, trnA-Trp). Besides, tRNA gene trnA-Ser existed as quadra copies. Twenty two protein-coding genes were extracted from mt genomes of 16 species to construct the neighbour-joining phylogenetic tree (Figure 1). A neighbour-joining phylogenomic analysis showed that *Citrus sinensis* was closely related to *Brassica napus* and *Arabidopsis thaliana*, which also belong to malvids.

**Figure 1.**
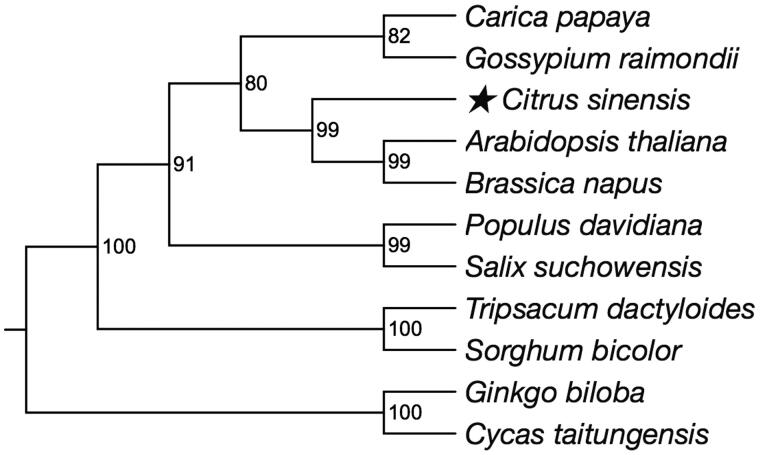
The neighbour-joining phylogenetic tree of *Citrus sinensis* was constructed with MEGA 7 with 1000 bootstrap replicates using 22 protein-coding genes of 11 species. All the sequences used could be available in the GenBank database: *Carica papaya* NC_012116; *Gossypium raimondii* NC_029998; *Citrus sinensis* NC_037463; *Arabidopsis thaliana* NC_001284; *Brassica napus* NC_008285; *Populus davidiana* NC_035157; *Salix suchowensis* NC_029317; *Tripsacum dactyloides* NC_008362; *Sorghum bicolor* NC_008360; *Ginkgo biloba* NC_027976; *Cycas taitungensis* NC_010303.
